# Biomechanical comparison of spinal column shortening - a finite element study

**DOI:** 10.1186/s12891-022-06047-5

**Published:** 2022-12-23

**Authors:** Jincheng Wu, Ye Han, Hanpeng Xu, Dongmei Yang, Wangqiang Wen, Haoxiang Xu, Jun Miao

**Affiliations:** 1 Department of Spine Surgery, Tianjin Hospital, Tianjin University, Jiefangnanlu 406, Hexi District, Tianjin, China; 2 The Affiliated Hospital of Hebei University, Baoding City, Hebei China; 3 Southern Medical University, Guangzhou City, China; 4 The First Affiliated Hospital of Hainan Medical University, Haikou City, Hainan, China; 5 The Second People’s Hospital of Hefei, Hefei, Anhui, China

**Keywords:** Spinal shortening, Total en bloc spondylectomy (TES), Spinal cord injury, Finite element analysis

## Abstract

**Background:**

At present, research on spinal shortening is mainly focused on the safe distance of spinal shortening and the mechanism of spinal cord injury, but there is no research on the biomechanical characteristics of different shortening distances. The purpose of this study was to study the biomechanical characteristics of spine and internal fixation instruments at different shortening distances by the finite element (FE) method.

**Methods:**

An FE model of lumbar L1-S was established and referred to the previous in vitro experiments to verify the rationality of the model by verifying the Intradiscal pressure (IDP) and the range of motion (ROM) of the motion segment. Five element models of spinal shortening were designed under the safe distance of spinal shortening, and the entire L3 vertebra and both the upper and lower intervertebral discs were resected. Model A was not shortened, while models B-E were shortened by 10%, 20%, 30% and 50% of the vertebral body, respectively. Constraining the ROM of the sacrum in all directions, a 7.5 N ·m moment and 280 N follower load were applied on the L1 vertebra to simulate the motion of the lumbar vertebrae in three planes. The ROM of the operated segments, the Von Mises stress (VMS) of the screw-rod system, the VMS of the upper endplate at the interface between the titanium cage and the L4 vertebral body, and the ROM and the IDP of the adjacent segment (L5/S) were recorded and analysed.

**Results:**

All surgical models showed good stability at the operated segments (L1-5), with the greatest constraint in posterior extension (99.3-99.7%), followed by left-right bending (97.9-98.7%), and the least constraint in left-right rotation (84.9-86.3%) compared with the intact model. The VMS of the screw-rod system and the ROM and IDP of the distal adjacent segments of models A-E showed an increasing trend, in which the VMS of the screw-rod system of model E was the highest under flexion (172.5 MPa). The VMS of the endplate at the interface between the cage and L4 upper endplate of models A-E decreased gradually, and these trend were the most obvious in flexion, which were 3.03, 2.95, 2.83, 2.78, and 2.61 times that of the intact model, respectively.

**Conclusion:**

When performing total vertebrae resection and correcting the spinal deformity, if the corrected spine has met our needs, the distance of spinal shortening should be minimized to prevent spinal cord injury, fracture of internal fixations and adjacent segment disease (ASD).

## Introduction

Since MacEwen et al. reported a series of neurological symptoms in 74 patients with spinal deformities after spinal orthopedic surgery in 1975, surgeons have begun to realize that excessive stretch of the spinal cord may cause damage to the nervous system [1]. Since then After that, surgeons began to consciously pay attention to the changes in spinal cord tension during the operation. Heish et al. found that in 1995 there was the first report in the literature that shortening the spine could be used to treat tethered cord syndrome [2]. After that, with the development of technology, spinal shortening technology has been more widely used. The spinal shortening technique is often used in traumatic or nontraumatic diseases, such as vertebral fracture with dislocation, spinal deformity, primary or secondary spinal malignant tumours, degenerative and infectious lesions [3–6]. At present, successful application of spinal shortening has been reported in various clinical fields. For example, Li et al. [7] have reported that 12 patients with scoliosis due to ankylosing spondylitis who underwent spinal shortening without postoperative neurological damage. Yoshioka et al. [8] reported 26 patients who underwent multi-segmental spinal resection for spinal tumors. Spinal shortening may provide good stability for multilevel TES. Shi et al. [9] reported a comparative study of 32 patients with spinal fractures who underwent spinal shortening surgery, and the postoperative clinical symptoms were significantly reduced. Mehdian et al. [10] reported that spinal shortening was performed in 8 patients with severe adolescent isthmic spondylolisthesis and achieved good results.

The spinal cord, which is segmentally tethered to the spine through the spinal cord tether and nerve root, is relaxed due to the decrease in tension caused by the shortening of the spinal column, but with the increase in the shortening distance, many neurological complications will occur [11]. Therefore, many studies have focused on the safe distance of spinal cord shortening and the mechanism of spinal cord injury caused by spinal column shortening. Many experiments have shown that when spinal shortening exceeds the safe distance limit, the spinal cord will kink, and changes in the spinal cord blood supply, blood barrier and spinal nerve root angle will damage the spinal cord [11–14], resulting in a series of neurological complications that seriously affect people’s quality of life. Recent studies have shown that spinal cord injury will be caused when the spinal column shortens more than 1/2 of the vertebral body height, leading to irreversible injury of somatosensory evoked potential (SSEP) and spinal cord blood flow (SCBF), resulting in neurological dysfunction [13]. And vertebral resection is performed in patients with a relatively good prognosis, so long-term postoperative stability is particularly important [15]. Because vertebral resection will cause spinal instability, auxiliary instrumentation is needed in the reconstruction of the spine. Postoperative instrument failure, bone non-union and cage subsidence may increase the possibility of secondary operation and affect the prognosis of patients. Jones et al. [16] reported that up to 44% of cancer patients treated with preoperative high-intensity radiation required secondary surgery for non-union after vertebrectomy. In addition, Matsumoto et al. [17] reported that about 40% of the patients failed internal fixation in the late stage after TES, including rod fracture, screw loosening, endplate fracture caused by cage subsidence and so on. Due to the high risk of the spinal shortening technique, it is important to have a better interbody fusion rate and a lower risk of screw-rod system fracture and cage subsidence without spinal cord injury.

However, to the best of our knowledge, there have been no finite element studies related to spinal shortening. Therefore, this study intended to evaluate the biomechanical characteristics of different shortening distances of the spine at a safe distance by finite element analysis.

## Materials and methods

### Intact FE model

Data from the L1-S lumbar spine FE model were collected from a healthy adult male volunteer (24 years old, weight 67 kg, height 173 cm). The volunteer had no previous history of trauma or fracture. Any spinal diseases were excluded by clinical imaging examination to establish a normal intact FE model. The volunteer was recruited by the Spinal Surgery Department of Tianjin Hospital and signed informed consent forms in accordance with the relevant regulations, which were submitted to the Ethics Committee for approval. A 64-slice spiral computed tomography scanner (GE, Siemens Sensation 16 Slice, Germany) was used to obtain the tomographic image data of the L1-S vertebrae with a 0.625 mm interslice interval in DICOM format. The image data were imported into Mimics 20.0 (Materialise, Belgium) to create a 3D surface model of the L1-S vertebrae and then into 3-Matic 12.0 software (Materialise lnc.) in STL format to perform wrapping and smoothing operations, remove excess triangular patches, and initially establish the structure of intervertebral disc and nucleus pulposus for exporting into Geomagic Studio 12.0 (Geomagic, Cary, NC, USA). After smoothing and accurate surface processing, the model was imported into Hypermesh 2017 (Altair Engineering, Troy, MI, USA) for mesh division and ligament construction and finally into Abaqus 2019 (Simulia, Johnston, RI, USA) for model assembly, material property definition and finite element analysis.

As shown in 1, a three-dimensional FE model of the normal L1-S lumbar vertebrae was constructed. The intervertebral disc is composed of the annulus ground substance, nucleus pulposus, annulus fibres and cartilaginous endplate, of which the nucleus pulposus accounts for 43% of the total disc [18]. Ligaments were simulated by using a tension-only truss element [19], and five layers of fibres were constructed from inside to outside and embedded into the annulus ground substance at an inclination of ± 30°. The elastic strength of the annulus fibres increased proportionally from the innermost (360 MPa) to the outermost fibres (550 MPa) [20–22]. Each vertebra was divided into cortical, cancellous and posterior bone structures, in which the thicknesses of cortical bone, endplate and articular cartilage were 1 mm, 0.5 mm and 0.2 mm, respectively [23, 24]. Facet contact surfaces were defined as surface-to-surface contacts with a friction coefficient of 0.1. The mesh convergence of the intact L1-S model was tested, which contained 1,489,577 elements and 370,061 nodes. The material properties were defined according to the previously reported literature, as shown in Table 1 [20, 23, 25, 26].

**Fig. 1:**
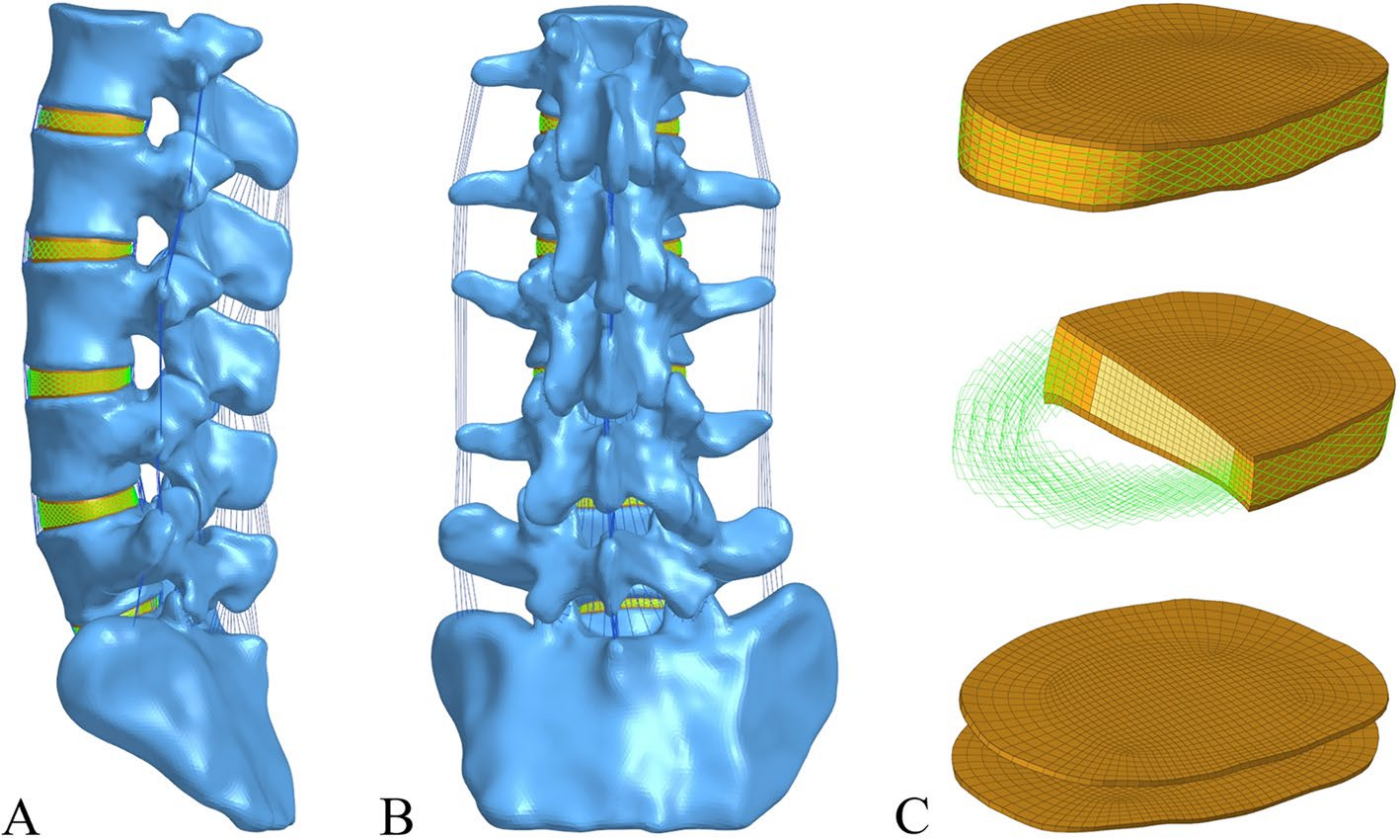
FE model of the intact L1-S vertebrae in the present study (**A**) Lateral view (**B**) Posterior view (**C**) Structure of the intact intervertebral disc

**Table 1:** Material properties used by finite element model

Component	Element type	Young’s modulus (MPa)	Poisson ratio	Cross-sectional area (mm^2^)
Vertebra				
Cortical bone	C3D4	12,000	0.3	
Cancellous bone	C3D4	100	0.2	
Posterior element	C3D4	3500	0.25	
Sacrum	C3D4	5000	0.2	
Facet	C3D4	11	0.2	
Disc				
Endplate	C3D8R	24	0.4	
Nucleus pulpous	C3D8RH	1	0.49	
Annulus ground substance	C3D8RH	2	0.45	
Annulus fibre	T3D2	360–550		0.15
Ligaments				
ALL	T3D2	7.8		63.7
PLL	T3D2	10		20
LF	T3D2	15		40
CL	T3D2	7.5		30
ISL	T3D2	10		40
SSL	T3D2	8		30
ITL	T3D2	10		1.8
Implants	C3D4	110,000	0.3	

*ALL* Anterior longitudinal ligament, *PLL* Posterior longitudinal ligament, *LF* Ligamentum flavum, *CL* Capsular ligament; *ISL* Interspinous ligament, *SSL* Supraspinal ligament; *ITL* Intertransverse ligament

### Model simulation

**Fig. 2:**
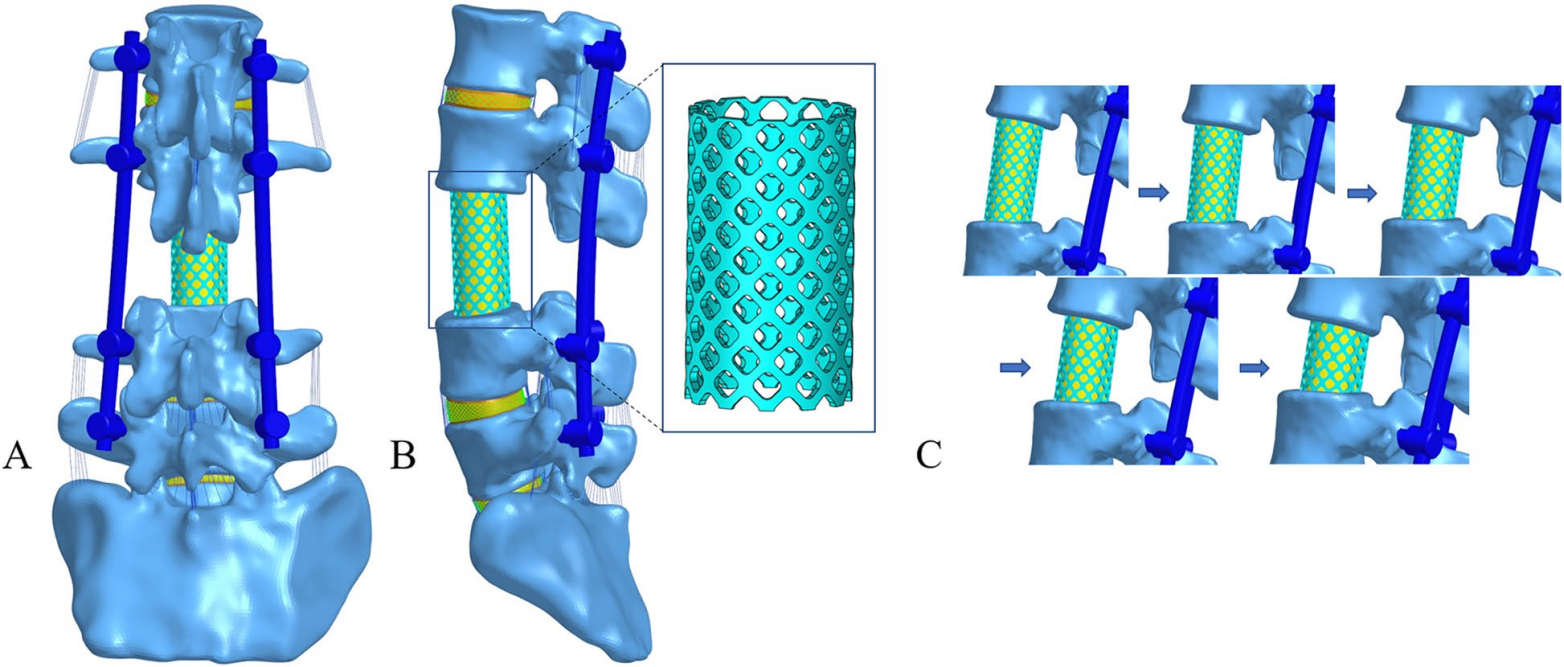
Surgical FE model fixed with implants. **A** Posterior view (**B**) Lateral view (**C**) Surgical FE models of the different shortening distances of the spine

As shown in Fig. 2, five models of spinal shortening were constructed in this study. Total en bloc spondylectomy (TES) removed the entire L3 vertebra and L2/3 and L3/4 intervertebral discs and moved the L1-2 segment downwards along the curvature of the lumbar vertebrae to simulate spinal shortening. Models A-E shortened the distances of 0%, 10%, 20%, 30% and 50% of the removed structure, respectively, in which titanium cages were placed and the two vertebrae adjacent to L3 were fixed with traditional trajectory screws (TTs). The diameter of the TT is 6.5 mm, and the length is 45 mm, while the diameter of the titanium cage is 18 mm, and the thickness is 1 mm.

### FE model validation

To validate the rationality of the model, the in vitro verification method of Renner et al. was implemented [27], in which the bottom of the sacrum was constrained in all degrees of freedom, and the motion of the spine in the sagittal, coronal and transverse planes was defined as flexion and extension and lateral bending and axial rotation, respectively. Four pure moments (flexion: 8 N·m, extension 6 N·m, lateral bending ± 6 N·m, rotation ± 4 N·m) were applied to the centre of the upper surface of the L1 vertebra, and the ROM of each segment was measured and compared with the in vitro study. In addition, referring to the previous in vitro experimental study by Brinckmann et al., the L4/5 IDP was measured by applying a gradually increasing compression force [28, 29].

### Boundary and loading conditions

The boundary conditions and loads of the FE model were loaded in ABAQUS software. In all the FE models, the bottom of the sacrum was constrained in all degrees of freedom, and a compressive preload of 280 N was applied on the upper surface of the L1 vertebra to simulate part of the body weight of the lumbar vertebrae [23, 30]. A pure moment of 7.5 N·m was applied to simulate flexion, extension, lateral bending and axial rotation.

### Assessment indexes

In this study, the biomechanical characteristics of the different surgical methods were analysed by measuring and calculating the ROM of operated segments, the VMS of the screw-rod system, the VMS at the interface between the titanium cage and the L4 upper endplate, and the ROM and IDP of the distal adjacent segment (L5/S).

## Results

### FE model validation

**Fig. 3:**
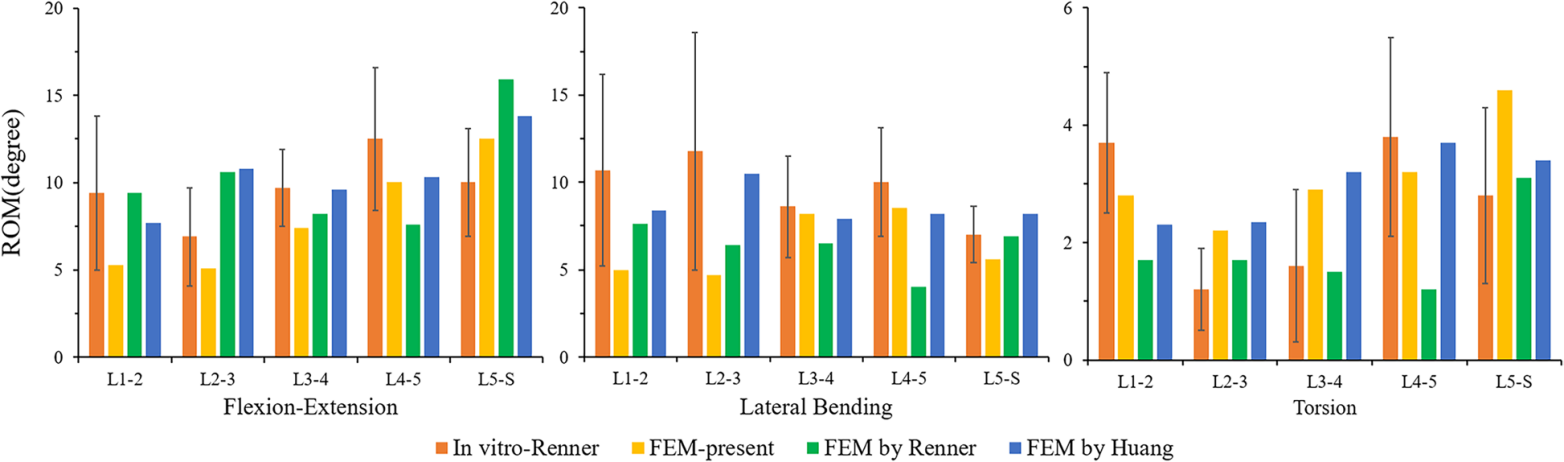
Comparison of the ROM of each motion segment between the current and previous studies

**Fig. 4:**
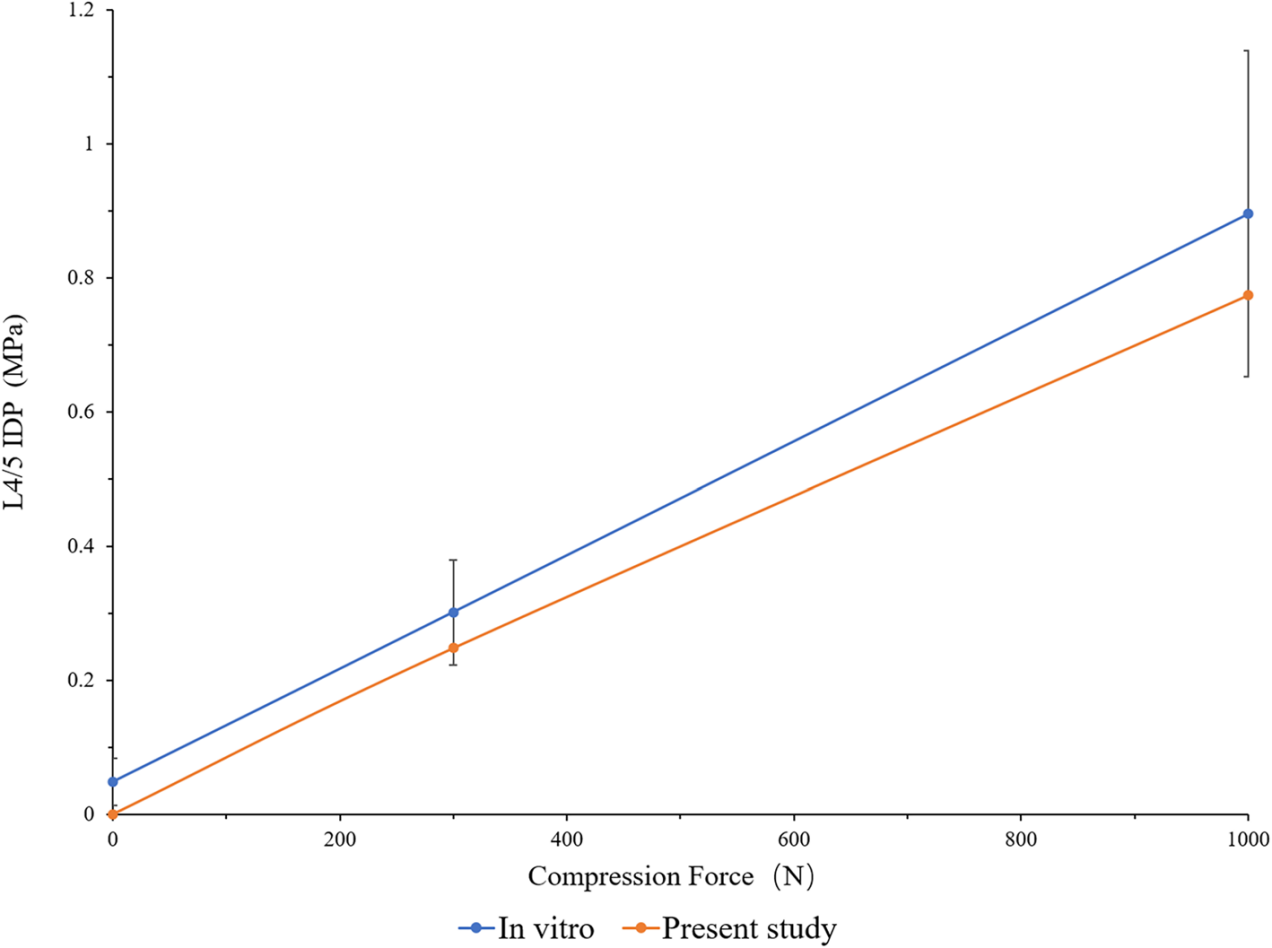
The IDP of L4/5 under the different compression loads (comparison with Brinckmann and Grootenboer, et al.)

In this study, the rationality of the FE model was verified by referring to the experimental method reported by Renner et al. By applying the same load and boundary conditions, the ROM of each vertebral segment of L1-S and the IDP of L4/5 were measured and compared with previous research results [26–28]. The results are shown in Figs. 3 and 4. The measured ROM of each vertebral segment was in good agreement with previous in vitro experiments and finite element studies. Under the increasing compression load, the L4/5 IDP value also had the same increasing trend. Therefore, we believe that the FE model of this study was effective for the following research.

### The ROM of the operated segment

**Fig. 5:**
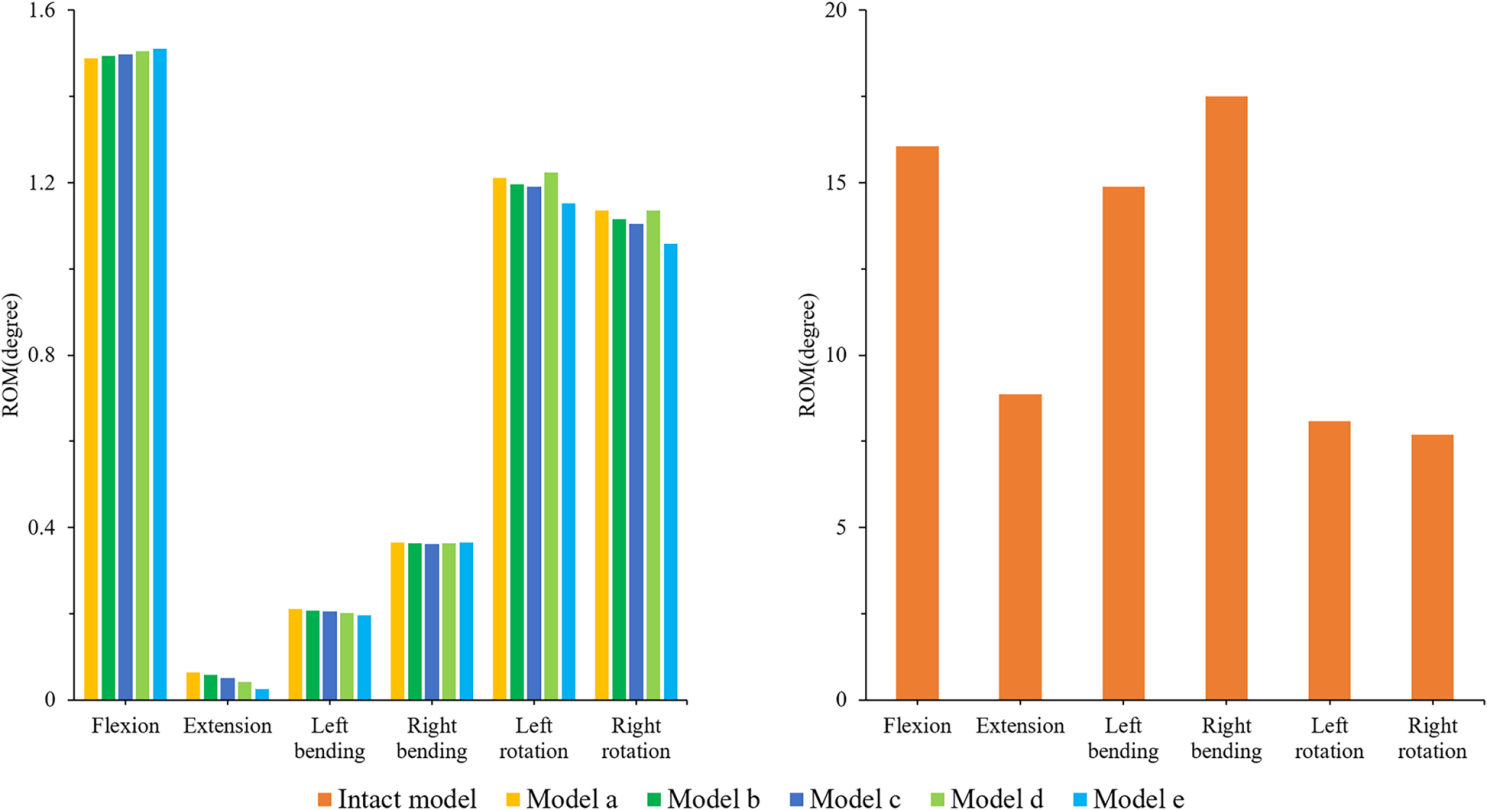
Comparison of the ROM between the intact and surgical FE models at the fusion segment (L1/5)

As shown in Fig. 5, the ROM of the operated segment (L1-5) of all the surgical models was significantly less than that of the intact model in all directions of movement, and the fixation device provided a good fixation effect in the operated segment. Among them, the fixture provided the best stability in the extension, which was the most restricted compared to the intact model (99.3-99.7%), followed by left-right bending (97.9-98.7%), and the least restriction in left-right rotation (84.9-86.3%). In terms of flexion, the operated segment motion showed a slight upwards trend in models A-E, but the difference between each model was less than 0.02 degrees. Therefore, this trend was not obvious. Under other loading conditions, the situation was reversed; model E had greater structural stiffness, and the fixture provided better stability.

### ROM of adjacent segment (L5/S)

**Fig. 6:**
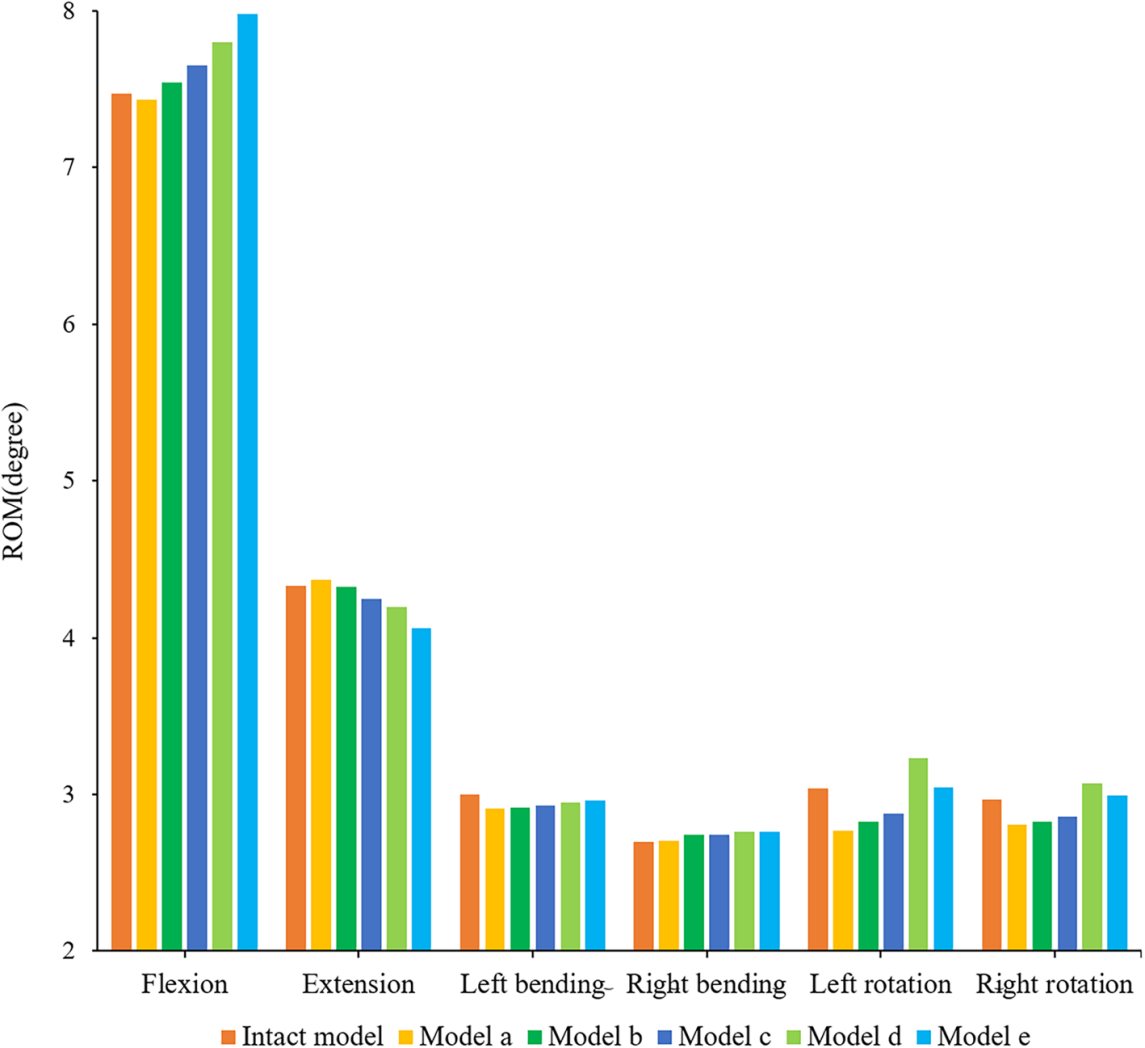
Comparison of the ROM between the different FE models at the adjacent motion segment (L5/S)

The ROM of L5/S for the different models under loading conditions is shown in Fig. 6. The motion trend of model L5/S in the sagittal plane was the opposite. Under the condition of flexion, the ROM of L5/S increased when increasing the shortening distance. Although the ROM of model A was smaller than that of the intact model, the difference between them was not significant (less than 0.1°). In terms of extension, the ROM of L5/S decreased with increasing spinal shortening distance (the maximum difference was 0.3°). In terms of coronal plane activity, there was no significant difference in the ROM of each model (less than 0.1°), and the use of the fixed device did not cause a significant change in the ROM of L5/S. In terms of cross-sectional activity, although the ROM between the models at L5/S tended to increase, the ROM of model D was the largest, with left-rotation and right-rotation larger than model E by 0.2° and 0.1°, respectively.

### IDP of adjacent segment (L5/S)

**Fig. 7:**
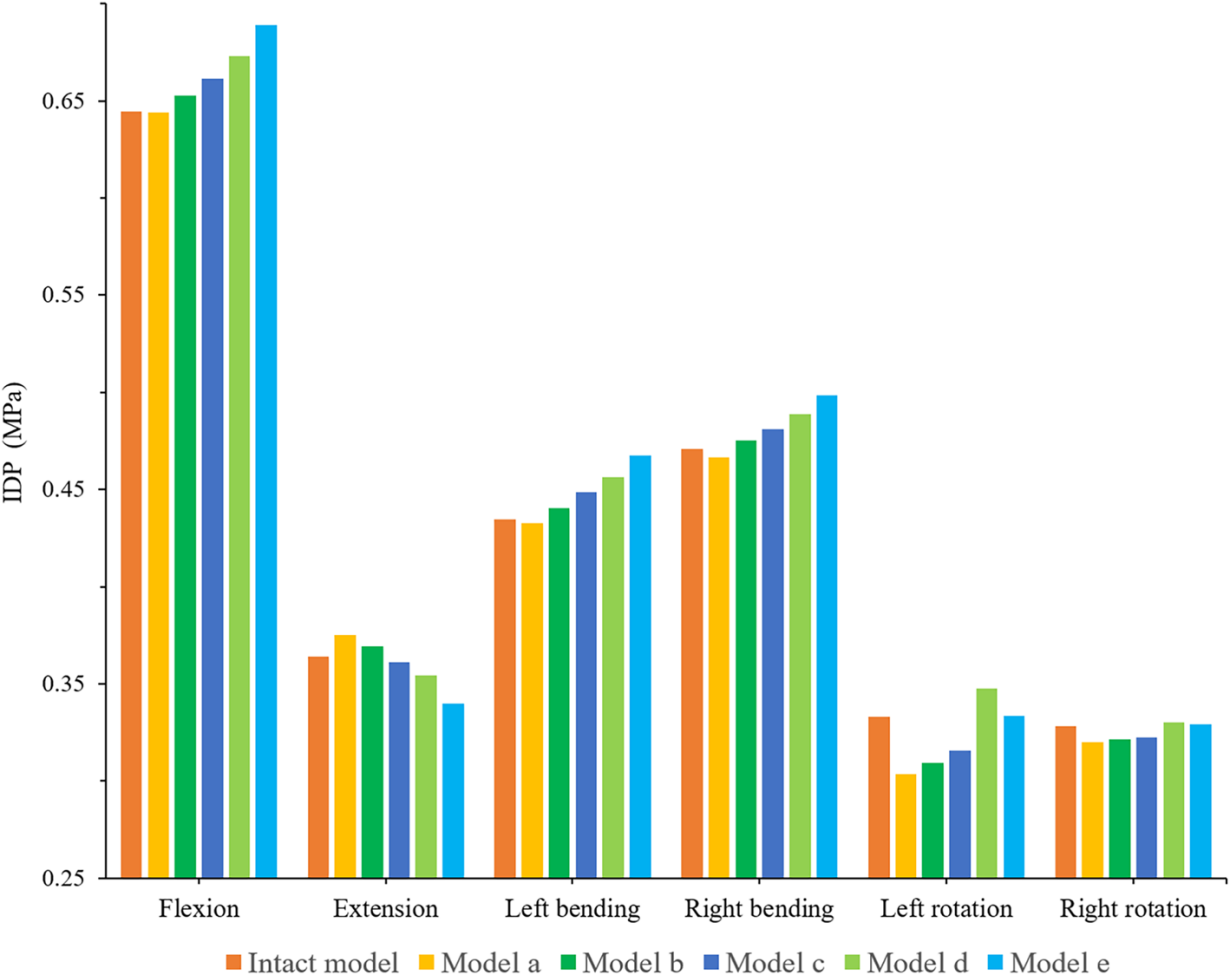
Comparison of the IDP between the different FE models at the adjacent motion segment (L5/S)

**Fig. 8:**
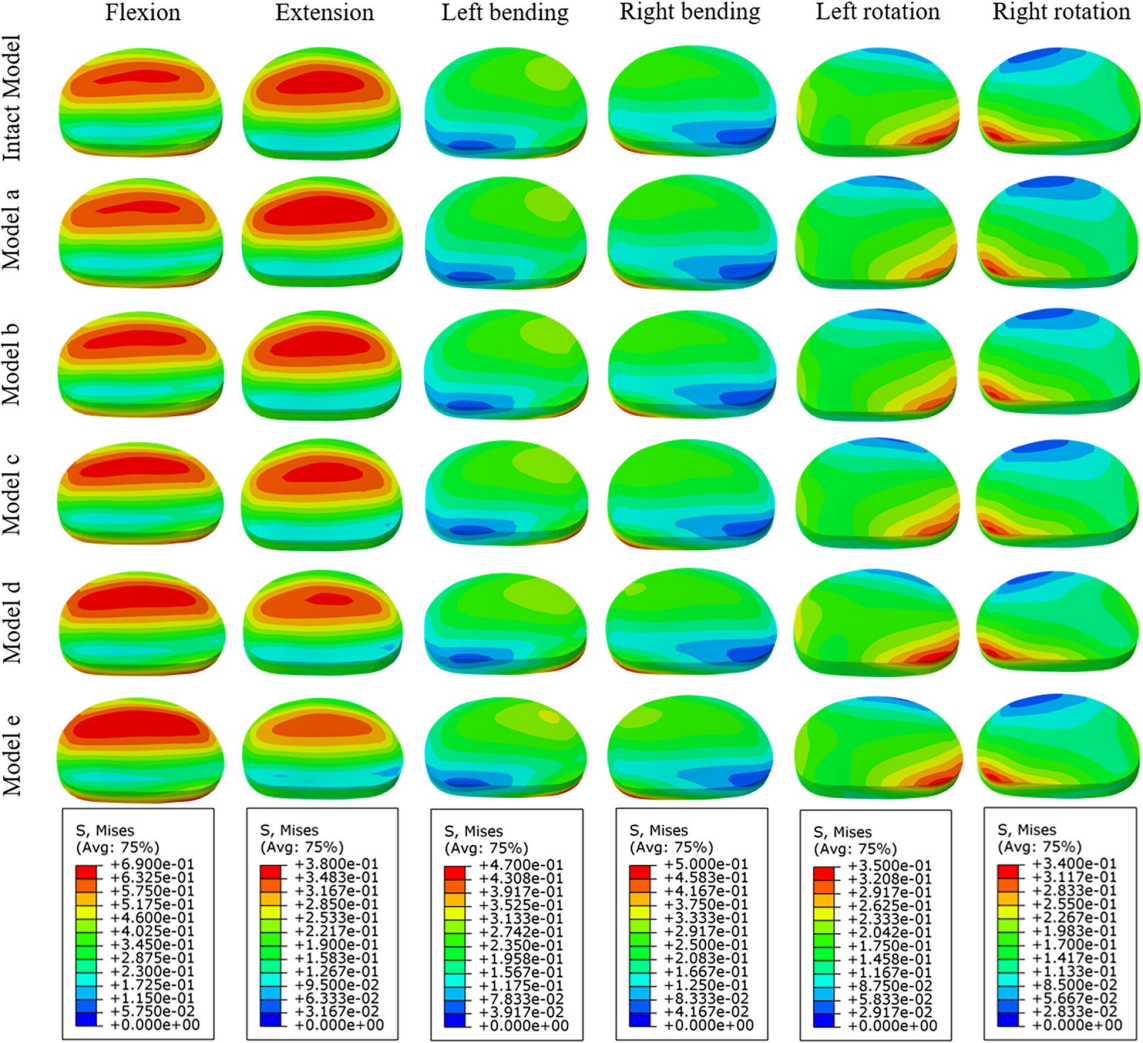
Von Mises stress (MPa) distribution of the L5/S IDP for the different FE models

The IDP trend and stress distribution of L5/S under all loading conditions of the different models are shown in Figs. 7 and 8. The surgical models had a consistent trend under other loading conditions except extension. With the increase in shortening distance, the IDP of L5/S increased, but the opposite was true under extension. In flexion, the IDP of model A was the smallest, but the difference between model A and the intact model was not significant (less than 0.01 Models B-E increased by 1.3%, 2.7%, 4.5% and 7.0% compared with model A, respectively. In extension, model A had the largest IDP, which was 0.38 MPa. The IDPs of models A-D were 10.5%, 8.7%, 6.3% and 4.3% higher than that of model E, respectively. In terms of lateral bending, the IDP of model E was the highest. The IDP of model E were 1.08, 1.06, 1.04 and 1.02 times those of model A-D in the left bending direction and 1.07, 1.05, 1.04, and 1.02 times those of model A-D in the right bending direction, respectively. In terms of rotation, the IDP of model D was greater than that of all the models, and with the increase in spinal shortening distance, the IDPs of models D and E were larger than that of the intact model. The IDP of model D was 1.15, 1.12, 1.10, and 1.04 times that of models A-E in the left rotation, while the IDP of model D was 1.03, 1.03, 1.02, and 1.01 times that of models A-E in the right rotation, respectively.

### Peak VMS of the endplate at the interface between the titanium cage and L4 upper endplate

**Fig. 9:**
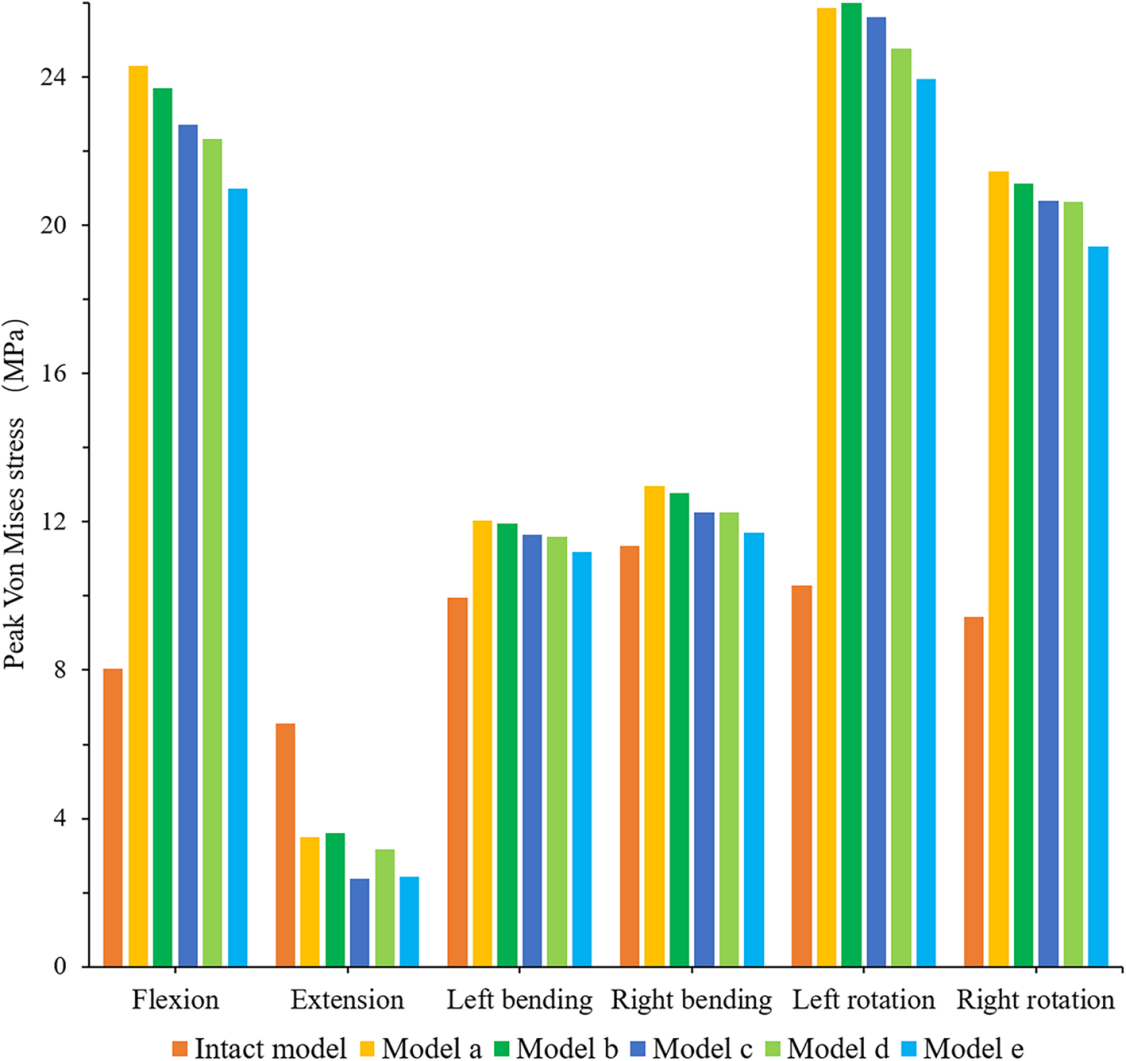
Comparison of maximum von Mises stress (MPa) of the endplate at the interface between the titanium cage and L4 upper endplate for the different FE models

**Fig. 10:**
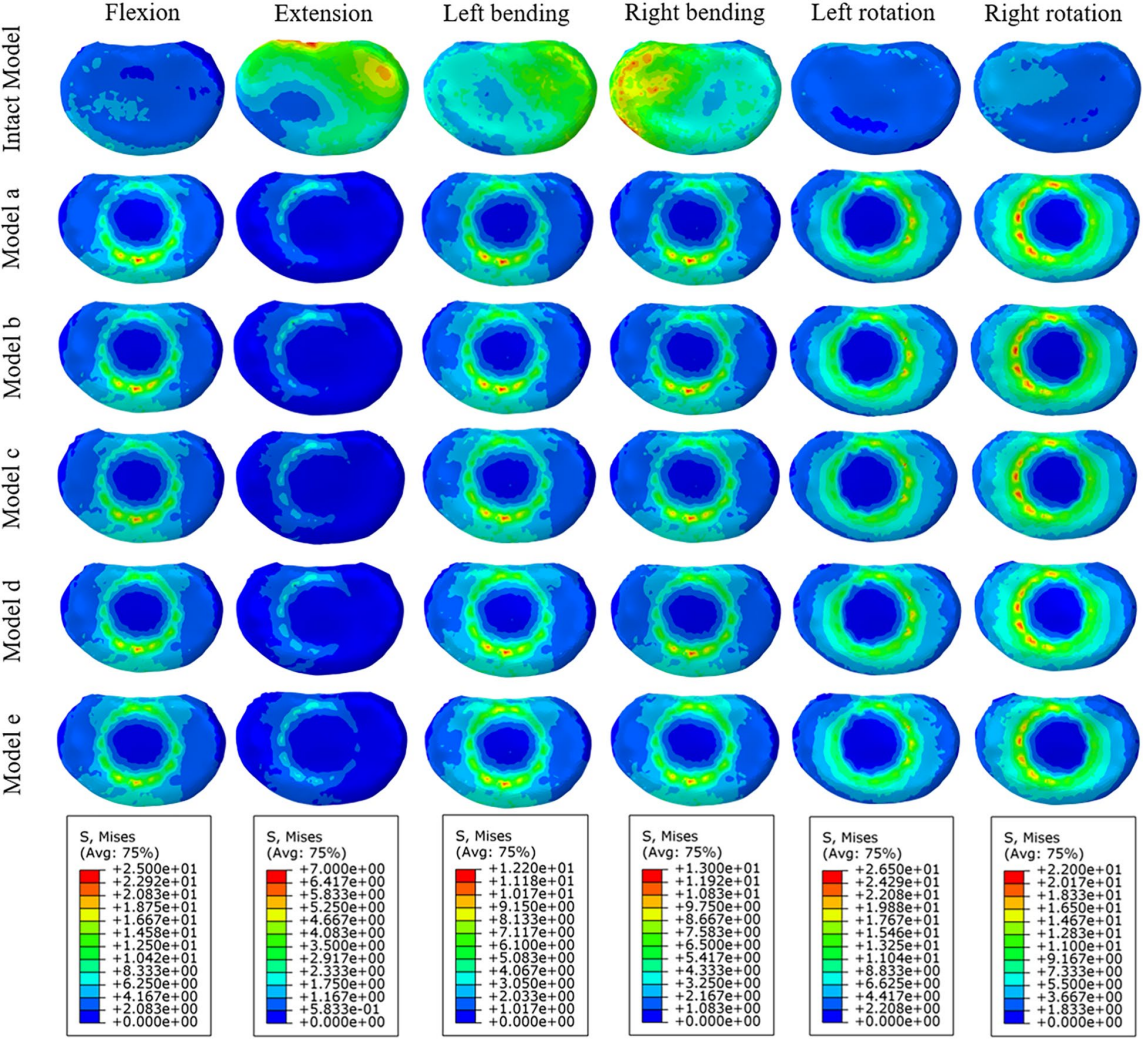
Von Mises stress (MPa) distribution of the endplate at the interface between the titanium cage and L4 upper endplate for the different FE models

As shown in Fig. 9, the peak stress of the endplate at the titanium cage-L4 upper endplate interface in all the surgical models was larger than that of the intact model, and it showed a good trend except for extension. The trend of gradual decrease of stress in models A-E was the most obvious in flexion, and the stresses of models A-E were 3.03, 2.95, 2.83, 2.78 and 2.61 times higher than that of the intact model, respectively. The maximum endplate stresses of A-E were 1.21, 1.20, 1.17, 1.16 and 1.12 times that of the intact model in left bending and 1.14, 1.12, 1.08, 1.08 and 1.03 times that in right bending, respectively. In terms of rotation, the maximum endplate stresses of models A-E were 2.52, 2.53, 2.49, 2.41 and 2.33 times that of the intact model in left rotation and 2.27, 2.24, 2.19, 2.19 and 2.06 times that in right rotation, respectively. Under all loading conditions, the stress distribution of the endplate on the L4 vertebral body of each model is shown in Fig. 10.

### Peak stress of rods

**Fig. 11:**
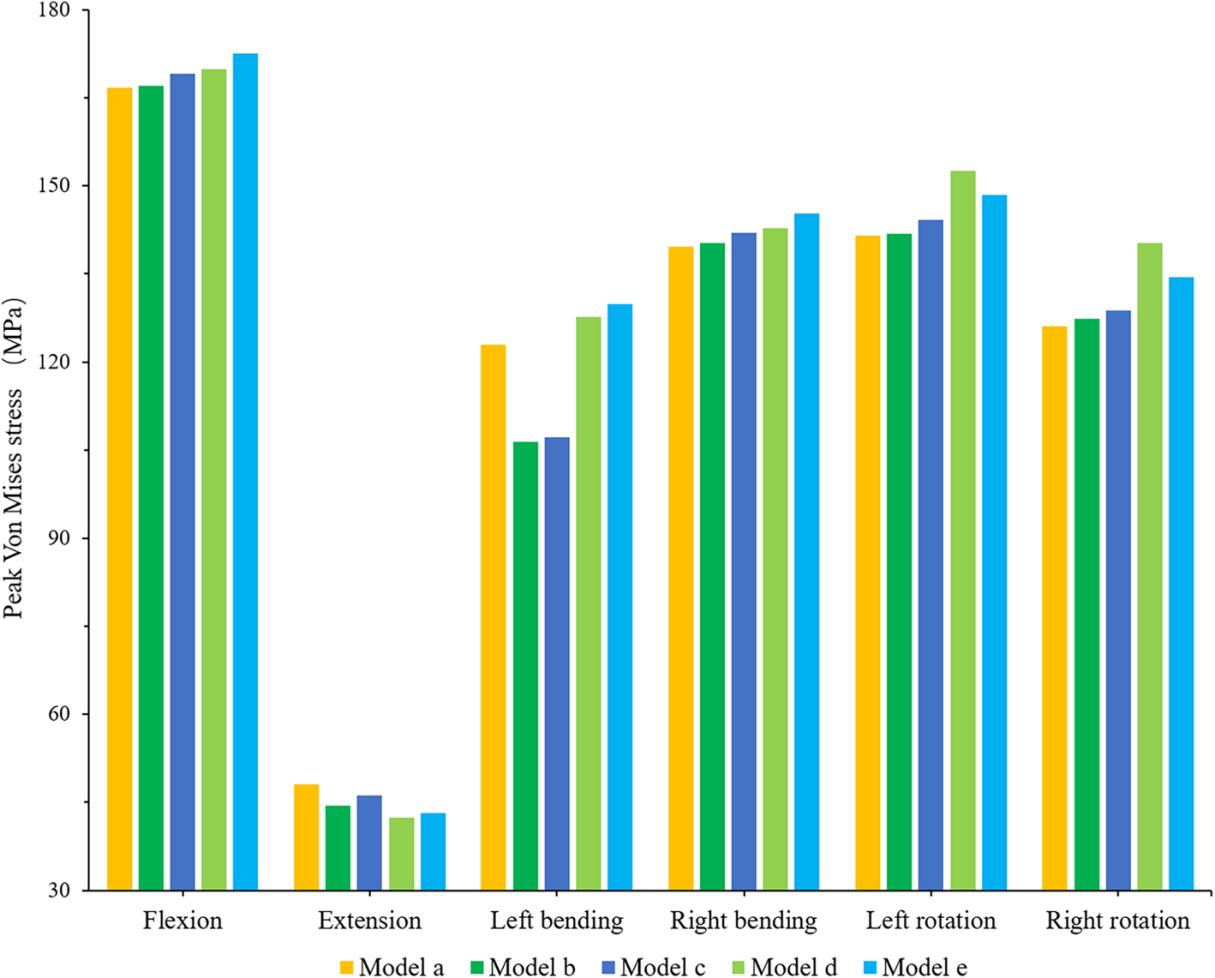
Comparison of the maximum von Mises stress (MPa) of the rods between the different FE models

**Fig. 12:**
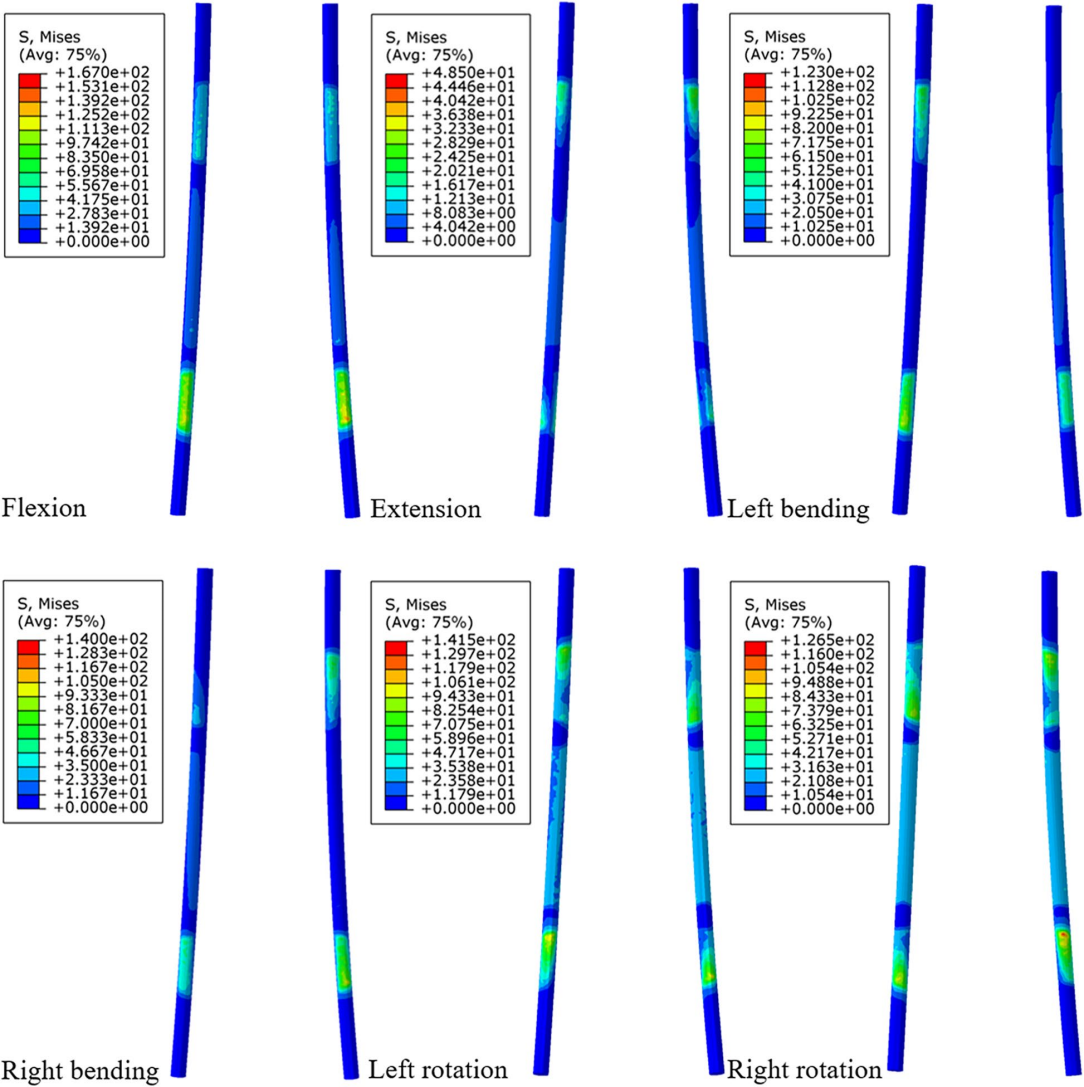
Von Mises stress (MPa) distribution of the rods for the model A under each activity

The maximum VMS trend of the posterior rods of each surgical model and the stress distribution under flexion are shown in Figs. 11 and 12. The VMS of rods in the other directions of movement changed well with the increase in spinal shortening distance except left bending. For example, the VMS of rods of models A-E showed an increasing trend in flexion, rotation and right bending, in which this trend was the most obvious in rotation, and the VMS of model D was significantly higher than that of the other models (the difference between model A and model D was 11.1 MPa in the left-rotation and 13.8 MPa in right rotation). Although the VMS of rods of models B and C was much lower than that of the other models in left bending, the stresses of models D and E were still larger than that of the other models. The stress of model E was the highest in flexion and right inclination, which was only different from model A by 5.8 MPa and 5.6 MPa, respectively. The VMS of rods was the smallest in extension, and generally speaking, its change trend decreased with increasing shortening distance. Although the VMS of rods of model C was larger than that of model B, it was only different from model B by 1.7.

## Discussion

The spinal shortening technique is often used in congenital spinal deformities, benign and malignant invasive tumours, spinal fracture and dislocation, tethered cord syndrome and other diseases [13, 31–33]. Among them, pedicle subtraction osteotomy or Smith-Peterson osteotomy was considered safe because of the smaller osteotomy range, smaller spinal shortening, and bone-bone interface contact to improve the fusion rate and stability [34]. However, for patients with tumour invasion, fracture and dislocation and rigid spinal deformity, TES often needs to be cured [7, 35]. Due to the possibility of cage fusion rate, fusion segmental stability, screw-rod fracture, and adjacent segmental disease after spinal surgery, a superior fixation method can bring long-term benefits to patients on the premise of fewer postoperative neurological complications. Although there are many reports about instrument failure after TES, there are few biomechanical studies on spinal reconstruction. For example, Yoshioka et al. [8] reported that 11 of 22 surgical patients had cage subsidence and 1 had rod breakage. Shinmura et al. [36] reported that 26 of the 61 patients who underwent TES surgery from 2010 to 2015 had instrument failure. Bone non-union and instrument failure would lead to neurological dysfunction, decline of quality of life and increase the burden of patients. Therefore, it is particularly important to study the biomechanical characteristics of each tissue structure after spinal shortening to provide a better theoretical basis for surgeons and to reduce the rate of postoperative revision. Recent experimental studies suggested that spinal shortening over 1/2 of the vertebral body can lead to serious postoperative complications [13]. Therefore, this study designed five models within the safe distance of spinal shortening, with models A-E shortening the vertebral body by 0%, 10%, 20%, 30% and 50% height, respectively, and analysed the biomechanical characteristics under different spinal shortening lengths.

Then, we evaluated the ability of the model to resist the motion of the operated segments by using the structural stiffness of the model. In this study, the structural stiffness of models A-E increased significantly and had a similar trend. Because the biomechanical assessment of intervertebral fusion was not possible, successful intervertebral fusion was considered according to the FDA definition of bridging trabecular bone between fusion segments, a translational activity less than 3 mm, and an ROM less than 5 degrees [37]. Liang et al. thought that the difference was considered significant when the results differed by more than 20% [19, 38]. In this study, the ROM of the fusion segments of models A-E were less than 5° in all directions, indicating that intervertebral fusion was successful. The results of our study showed that, regardless of the fixation method, the restriction of internal fixation devices on the model was most significant in extension, followed by lateral bending, and minimal in rotation, which was similar to the results of previous biomechanical studies [39, 40]. This may be because the posterior structures of the adjacent vertebrae, including the lamina and facet joints and ligaments, were not damaged during the operation, so they can still play a role in maintaining the stability of the vertebrae in future activities. In this study, although the ROM of models A-E tended to increase slightly in flexion, the maximum difference in ROM was not more than 0.02°, while in other directions, the ROM tended to decrease with increasing shortening distance, which was more obvious in extension and rotation. Although it is a kind of static analysis result, it still showed an overall trend. Among them, the model with a 50% reduction in vertebral height provided only 6% more stability in rotation than before spinal shortening but 59% more stability in extension.

Spinal fusion surgery accelerates the onset of ASD by increasing intervertebral mobility and mechanical stress, resulting in severe back pain, radicular symptoms, or neurogenic intermittent claudication, which can seriously affect people’s daily lives [41–43]. It has been reported that the incidence of a second operation for ASD was 4% per year, 16.5% at 5 years, but up to 36.1% at 10 years [44]. In this study, the ROM and IDP of the adjacent segment (L5/S) showed good consistency among each model, and they reflected the motion state of the adjacent segment after the operation. In extension, the ROM and IDP of L5/S decreased slightly with increasing shortening distance, while the situation was opposite under other loading conditions. Although the ROM of each model was consistent under lateral bending, the change in IDP was more obvious than that in ROM. The IDP of model E differed from that of the intact model and model A by 6.7% and 7.4% in lateral bending, respectively. Although generally speaking, with an increase in the shortening distance, the ROM and IDP of L5/S of model A-E showed an increasing trend; they were not all larger than those of the intact model. Cho et al. reported that in their study, the risk of ASD in the proximal adjacent segment was the highest, which was higher than that of the distal adjacent segments [45], so there may be some possibility that the ROM of distal adjacent segments was smaller than the intact model in some loading conditions. However, the trend of ROM and IDP between models in this study can also give us an important hint.

In vertebral fusion, we should not only consider the possibility of bone graft nonfusion and adjacent segment disease but also consider the risk of vertebral bone destruction and cage subsidence at the interface between the cage and vertebral body. In this study, except for the extensional loading condition, the endplate stress of all surgical models at the interface between the cage and L4 upper endplate was greater than that of the intact model, and the stress showed a downwards trend in models A-E. The reason may be that the internal fixation and posterior structure of the vertebral body limit the extension movement of the spine, thus affecting the VMS of the endplate. It was previously reported that the failure strength of cortical bone was between 90 and 200 MPa [46]. Our study found that although the stress of the endplate increased in all surgical models as a whole, the result was much smaller than that of 90 MPa, within the range of failure strength of vertebral cortical bone. Because of the wide range of total vertebrae resection, it greatly affected the stability of the spine, which mainly depended on the internal fixation to maintain the stability of the spine. Sciubba et al. reported that in their study, the rod breaking rate of 23 patients who underwent TES was as high as 39.1% [47]. Our study found that although the stress trends in the extension of rods of models A-E were different from that of the other directions of activity, generally speaking, the stress of rods increased with an increasing shortening distance. However, in this study, the maximum stress (172.5 MPa) of rods under flexion was much lower than the yield strength of titanium alloy (825–895 MPa). In rotation, the stress of rods of model D was greater than that of model E. The possible reason was that with the increase in the shortening distance, the upper and lower articular processes of the adjacent vertebrae contact each other, thus limiting the movement between the vertebral body. As a result, the stress of the fixed rods was reduced.

Although the results of this study show that spinal shortening technique has certain advantages in the stability of fusion segments and the reduction of endplate stress, the spinal shortening technique still has certain limitations in clinical application. In children, the growth of non-fusion segments during the growth of the spine may reestablish the tension of the spinal cord, it may affect the annual 1 mm growth retardation of the spinal fusion segments, and even lead to crankshaft deformities [32]. Moreover, the operation of spinal shortening is relatively complex, the operation time is long, and the blood loss is large, and it is possible to undergo a second operation because of instrument failure reported by Aldave et al. [48, 49]. And there may be spinal cord injury caused by too long intraoperative shortening distance or too short shortening distance leading to treatment failure. We must consider the adverse consequences of these spine-shortening techniques when making surgical decisions for patients, and personalize the plan to maximize patient benefit compared with other surgical methods.

There were some limitations in this study. The finite element model data in this study were only based on a 24-year-old adult male, and this study was without statistical analysis, which is a common defect of finite element analysis. At the same time, this study simplified the finite element model, and the material properties of each structure were assumed to be isotropic, which cannot more accurately reflect the biomechanical changes of the lumbar structure. Second, previous studies on the mechanism of acute spinal shortening and spinal cord injury were often based on animal experiments, in which the selected segments were equivalent to the human thoracolumbar vertebrae or the middle and lower thoracic vertebrae to simulate the actual situation in the human body 11–13, 50]. The analysis results of this study were based on L1-S vertebrae, and the safe distance at the cauda equina level may be larger than that of the thoracolumbar segment. In addition, because of the risk of spinal shortening surgery, serious neurological complications may occur after operation, so the previous research results were based on animal experiments. Therefore, there is a lack of research data in the human body. However, biomechanics can simulate the physiological conditions and surgical process of normal people, thus avoiding unnecessary risks, but this study did not carry out biomechanical studies on the spinal cord and spinal nerves, which was another limitation of our study. In the future, we plan to conduct more reasonable and rigorous biomechanical studies to verify our results.

## Conclusion

The results of this study suggested that under the condition of ensuring the safety of the spinal cord, with the increase in the spinal shortening distance, it increased the stress of the internal rods and ROM and IDP of the adjacent distal segment, although it reduced the stress of the endplate at the interface between the titanium cage and L4 upper endplate and provided a slight advantage for the stability of the fusion segments. Therefore, when spinal correction meets our needs during surgery, the distance of spinal shortening should be reduced as much as possible to prevent spinal cord injury, internal rod fracture and the occurrence of ASD.

## Data Availability

The datasets used and/or analysed during the current study available from the corresponding author on reasonable request.

## Abbreviations

FE: Finite element
ROM: Ranges of motion
VMS: Von Mises stress
TTs: Traditional trajectory screws
IDP: Intradiscal pressure
ASD: Adjacent segment disease
TES: Total en bloc spondylectomy
SSEP: Somatosensory evoked potential
SCBF: Spinal cord blood flow

## References

[1] MacEwen GD, Bunnell WP, Sriram K (1975). Acute neurological complications in the treatment of scoliosis. A report of the Scoliosis Research Society. J Bone Joint Surg Am Vol.

[2] Hsieh PC (2010). Posterior vertebral column subtraction osteotomy for the treatment of tethered cord syndrome: review of the literature and clinical outcomes of all cases reported to date. Neurosurg Focus.

[3] Obeid I (2011). Total vertebrectomy and spine shortening in the management of Acute thoracic spine fracture dislocation. J Spin Disord Tech.

[4] Lorente A (2018). Vertebrectomía total y acortamiento vertebral en el manejo de luxación vertebral T12-L1: manejo con medios subóptimos. Neurocirugía.

[5] Gokcen B, Yilgor C, Alanay A (2014). Osteotomies/spinal column resection in paediatric deformity. Eur J Orthop Surg Traumatol.

[6] Oka S (2016). Total or partial vertebrectomy for lung cancer invading the spine. Annals Med Surg (2012).

[7] Li T (2020). A preliminary study of spinal cord blood flow during PVCR with spinal column shortening: a prospective clinic study in severe rigid scoliokyphosis patients. Medicine.

[8] Yoshioka K (2013). Clinical outcome of spinal reconstruction after total en bloc spondylectomy at 3 or more levels. Spine.

[9] Shi Z (2022). Posterior Injured vertebra column resection and spinal shortening for thoracolumbar fracture associated with severe spinal cord injury: a retrospective case-control observational study. Comput Intelligence Neurosci.

[10] Mehdian SH, Arun R (2011). A new three-stage spinal shortening procedure for reduction of severe adolescent isthmic spondylolisthesis: a case series with medium- to long-term follow-up. Spine.

[11] Kawahara N (2005). Influence of acute shortening on the spinal cord: an experimental study. Spine (Phila Pa 1976).

[12] Ji L (2013). Study on the safe range of shortening of the spinal cord in canine models. Spinal Cord.

[13] Ji L (2020). Safe range of shortening the middle thoracic spine, an experimental study in canine. Eur Spine J.

[14] Yang H (2020). Relationship between the laminectomy extension and spinal cord injury caused by acute spinal shortening: goat in vivo experiment. Eur Spine J.

[15] Tomita K (2001). Surgical strategy for spinal metastases. Spine (Phila Pa 1976).

[16] Jones M (2018). Total en bloc spondylectomy. J Spine Surg.

[17] Matsumoto M (2011). Late instrumentation failure after total en bloc spondylectomy. J Neurosurg Spine.

[18] Polikeit A (2003). Factors influencing stresses in the lumbar spine after the insertion of intervertebral cages: finite element analysis. Eur Spine J.

[19] Li C (2014). Treatment of unstable thoracolumbar fractures through short segment pedicle screw fixation techniques using pedicle fixation at the level of the fracture: a finite element analysis. PLoS ONE.

[20] Lu T, Lu Y (2019). Comparison of Biomechanical Performance among Posterolateral Fusion and Transforaminal, Extreme, and oblique lumbar Interbody Fusion: a finite element analysis. World Neurosurg.

[21] Schmidt H (2007). Application of a calibration method provides more realistic results for a finite element model of a lumbar spinal segment. Clin Biomech (Bristol Avon).

[22] Shin DS, Lee K, Kim D (2007). Biomechanical study of lumbar spine with dynamic stabilization device using finite element method. Comput Aided Des.

[23] Choi J, Shin D, Kim S (2017). Biomechanical Effects of the geometry of ball-and-Socket Artificial disc on lumbar spine. Spine.

[24] Kim H (2014). Biomechanical Analysis of Fusion Segment Rigidity upon stress at both the Fusion and adjacent segments: a comparison between unilateral and bilateral pedicle screw fixation. Yonsei Med J.

[25] Su Q (2020). Analysis and improvement of the three-column spinal theory. BMC Musculoskelet Disord.

[26] Huang Y (2016). Preserving posterior complex can prevent adjacent segment disease following posterior lumbar Interbody Fusion Surgeries: a finite element analysis. PLoS ONE.

[27] Renner SM (2007). Novel model to analyze the effect of a large compressive follower pre-load on range of motions in a lumbar spine. J Biomech.

[28] Brinckmann P, Grootenboer H (1991). Change of disc height, radial disc bulge, and intradiscal pressure from discectomy. An in vitro investigation on human lumbar discs. Spine (Phila Pa 1976).

[29] Dreischarf M (2014). Comparison of eight published static finite element models of the intact lumbar spine: predictive power of models improves when combined together. J Biomech.

[30] Shen H (2019). Biomechanical analysis of different lumbar interspinous process Devices: a finite element study. World Neurosurg.

[31] McVeigh LG, et al. Spinal column shortening for tethered cord syndrome: a systematic review and individual patient data meta-analysis. J Neurosurg Pediatr. 2022;29(6):624–33. 10.3171/2022.1.PEDS21503 35245903

[32] McVeigh LG (2021). Spinal column shortening for secondary tethered cord syndrome: radiographic, clinical, patient-reported, and urodynamic short-term outcomes. J Neurosurg Pediatr.

[33] Zhao Z (2021). Spinal-shortening process positively improves Associated Syringomyelia in patients with Scoliosis after single-stage spinal correction. World Neurosurg.

[34] Alemdaroğlu KB (2007). Morphometric effects of acute shortening of the spine: the kinking and the sliding of the cord, response of the spinal nerves. Eur Spine J.

[35] AlEissa SI, et al. Management of thoracic spine dislocation by total vertebrectomy and spine shortening: case report. 2020;6(1):80. 10.1038/s41394-020-00327-9 PMC7445242 32839430

[36] Shinmura K (2020). Revision surgery for instrumentation failure after total en bloc spondylectomy: a retrospective case series. BMC Musculoskelet Disord.

[37] Boustani HN (2012). Which postures are most suitable in assessing spinal fusion using radiostereometric analysis?. Clin Biomech Elsevier Ltd.

[38] Liang Y, et al. A finite element analysis on comparing the stability of different posterior fixation methods for thoracic total en bloc spondylectomy. 2020;15(1):314. 10.1186/s13018-020-01833-0 PMC7422552 32787876

[39] Tan Q (2021). Biomechanical comparison of four types of instrumentation constructs for revision surgery in lumbar adjacent segment disease: a finite element study. Comput Biol Med.

[40] Wang W (2019). Biomechanical effects of posterior pedicle fixation techniques on the adjacent segment for the treatment of thoracolumbar burst fractures: a biomechanical analysis. Comput Methods Biomech BioMed Eng.

[41] Louie PK (2019). Comparison of stand-alone lateral lumbar Interbody Fusion Versus Open Laminectomy and Posterolateral Instrumented Fusion in the treatment of adjacent segment Disease following previous lumbar Fusion surgery. Spine.

[42] Hekimoğlu M (2021). Adjacent segment disease (ASD) in Incidental Segmental Fused Vertebra and Comparison with the Effect of Stabilization Systems on ASD. Cureus.

[43] Song K (2011). Adjacent segment degenerative disease: is it due to disease progression or a fusion-associated phenomenon? Comparison between segments adjacent to the fused and non-fused segments. Eur Spine J.

[44] Louie PK (2020). Etiology-based classification of adjacent segment Disease following lumbar Spine Fusion. HSS J ®.

[45] Cho HJ (2021). The efficacy of lumbar Hybrid Fusion for the Prevention of adjacent segment disease: fact or artifact? A Meta-analysis. Clin Spine Surg.

[46] Liang Z (2020). Biomechanical evaluation of strategies for adjacent segment disease after lateral lumbar interbody fusion: is the extension of pedicle screws necessary?. BMC Musculoskelet Disord.

[47] Sciubba DM (2016). Total en bloc spondylectomy for locally aggressive and primary malignant tumors of the lumbar spine. Eur Spine J.

[48] Aldave G (2017). Spinal column shortening for tethered cord syndrome associated with myelomeningocele, lumbosacral lipoma, and lipomyelomeningocele in children and young adults. J Neurosurg Pediatr.

[49] Zhang C, et al. Spinal column shortening versus revision detethering for recurrent adult tethered cord syndrome: a preliminary comparison of perioperative and clinical outcomes. J Neurosurg Spine. 2020;32(6):958–64. 10.3171/2019.12.SPINE19659 32032960

[50] Shengli Huang LXYH. Electrophysiological monitoring techniques for spinal cord function in a canine model. Int J Clin Exp Med. 2018;11(6):5986–91.

